# Future Time Perspective and Bedtime Procrastination: The Mediating Role of Dual-Mode Self-Control and Problematic Smartphone Use

**DOI:** 10.3390/ijerph191610334

**Published:** 2022-08-19

**Authors:** Bo Mao, Shuai Chen, Mingchen Wei, Yali Luo, Yanling Liu

**Affiliations:** 1 Research Center of Mental Health Education, Faculty of Psychology, Southwest University, Chongqing 400715, China; 2 Center of Mental Health Education, Southwest University of Political Science & Law, Baosheng Avenue No. 301, Chongqing 401120, China

**Keywords:** future time perspective, bedtime procrastination, dual-mode of self-control, problematic smartphone use, temporal self-regulation theory

## Abstract

This study examined bedtime procrastination predictors and the development process concerning health behavior. Based on temporal self-regulation theory and the self-regulatory framework of time perspective, we examined the effects of future time perspective, dual-model of self-control, and problematic smartphone use on bedtime procrastination. Further, including the mediating role of dual-mode self-control and problematic smartphone use in the effects of future time perspective on bedtime procrastination among 3687 participants (38.73% male; *M*_age_ = 16.17 years, *SD* = 2.42, range = 11–23) Chinese students. The results showed that the future time perspective, dual-mode self-control, and problematic smartphone use had significant predictive effects on bedtime procrastination. Importantly, the negative effect of future time perspective on bedtime procrastination is mediated by the impulse system, control system, and problematic smartphone use separately and serially mediated by the impulse system and problematic smartphone use, rather than the control system and problematic smartphone use; these findings extend previous research on the contributing factors of bedtime procrastination and provide an empirical basis for promoting people to form healthy sleep habits.

## 1. Introduction

In the past 10 years, the Chinese population’s sleep time has been delayed by more than 2 h, and the sleep duration has been shortened by nearly 1.5 h [1]. One primary reason is bedtime procrastination, defined as “failing to go to bed at the intended time, while no external circumstances prevent a person from doing so” [2]. Many studies have demonstrated that bedtime procrastination is widespread in the general population and is associated with many adverse consequences, such as sleep problems (e.g., sleep insufficiency, poor sleep quality, and insomnia) [3,4,5]; further, it may also be associated with physical and psychological diseases (e.g., negative affect, depression, and anxiety symptoms) [6,7]. Therefore, it is necessary to identify the contributing factors and developmental processes of bedtime procrastination.

Temporal Self-regulation Theory (TST) is a healthy behavior theory based on the “intention-behavior” model; it emphasizes the effects of temporal, self-regulation capacity, and behavioral prepotency [8], which can help understand the occurrence of bedtime procrastination. The TST posits that connectedness beliefs (beliefs about the consistency of present behavior to later outcomes) and temporal valuations (values attached to temporally dispersed results) determine the behavioral intention and affect health behavior. Meanwhile, self-regulatory capacity and behavior prepotency can moderate the strength of the intention-behavior link and directly affect behavior. For example, bedtime procrastinators are still aware of the benefits of sleep. Therefore, they are eager to rest from sleep [9], which means they have connectedness beliefs for sleeping on time and resisting procrastination. Specifically, bedtime procrastination is more likely because of the weak sleep intention caused by low temporal valuations and the failure of behavior transformation driven by a lack of self-regulatory capacity and behavior prepotency; however, researchers have yet to examine it [10].

This study aimed to investigate the antecedents of bedtime procrastination drawing on TST, which takes future time perspective as the temporal valuation, dual-mode of self-control as self-regulatory capacity, and problematic smartphone use as behavior prepotency. The self-regulatory framework of time perspective postulates that self-regulatory ability and goal monitoring and operation can offer insight into the associations between future time perspective and people’s behavior [11]; moreover, dual-process models of addictive behavior and prior research have linked dual-mode self-control and problematic smartphone use [12]. Therefore, the current study also aimed to examine the mediating roles of dual-mode self-control and problematic smartphone use in the association between future time perspective and bedtime procrastination.

### 1.1. Future Time Perspective and Bedtime Procrastination

Future time perspective is an individual temporal difference defined as a general concern for and corresponding consideration of one’s future [13,14]. Individuals with a higher future time perspective fully consider the potential distant outcomes of their current behavior, pursue high-value goals, and guide and dominate personal behavior through future goals and rewards [15]. Numerous studies have demonstrated that the ability to foresee, anticipate, and plan for future outcomes is crucial for achievement, well-being, and health behaviors [11,13,16].

According to the TST, the temporal valuations of future behavior assessment based on behavioral value and perceived temporal proximity determine behavior intention and then affect health behavior [8]; this intertemporal assessment process largely depends on individuals’ future time sensitivity and subjective experience, the future time perspective. The benefits of regular sleep can only be manifested through long-term persistence. The contradiction between high values and low temporal proximity is highlighted, reducing temporal valuations, and decreasing sleep intention [10]; however, individuals with higher future time perspective have more significant value attachments to future goals [13] and shorten the perception of time delay with “future” as the reference point [17], which will reduce the value discount of future behavior. Therefore, they may give greater temporal valuations to sleep because of the high-value evaluation and perception of temporal proximity, which helps to improve sleep intention and resist bedtime procrastination.

Previous studies have linked future time perspective with procrastination, and health behavior also provides indirect evidence that future time perspective affects bedtime procrastination. For instance, empirical evidence and meta-analysis results indicate that future time perspective is negatively associated with procrastination [18,19]. Furthermore, multiple meta-analyses have demonstrated that future time perspective is positively related to health-promoting behavior (i.e., exercise behavior) [11,20,21] and the level of physical health (i.e., lower BMI) [11,13,16,21]. Further, it is negatively associated with health risk behavior (i.e., substance use) [11,13,20]. In conclusion, based on the viewpoints of TST and the relationships between future time perspective, procrastination, and health behavior, it is reasonable to postulate the first hypothesis:

**Hypothesis** **1** **(H1).** *Future time perspective is negatively correlated with bedtime procrastination.*

### 1.2. Dual-Mode of Self-Control as a Mediator

Self-control is defined as the capacity to alter one’s responses, especially to bring them into line with standards such as self and social expectations, and to support the pursuit of long-term goals [22]; this holds the essentiality to understanding the occurrence of health behavior [8] and procrastination [23]. Similarly, bedtime procrastination is most often considered a self-control failure caused by low self-control capacity [2,5,24,25] or self-control depletion [26].

However, compared to a single perspective, the dual-systems model posits that it is more reasonable to examine self-control from the perspective of impulse and control [27,28]. While the impulsive system is a process of an automatic reaction to external stimuli, affective and rewards oriented toward immediate pleasure, the control system is responsible for higher order mental operations which serve regulatory goals and long-term interests that complement the functions of the impulsive system. Self-control failure is caused by the impulsive system taking precedence over the control system; however, previous studies have generally described bedtime procrastination based on control systems and have mostly ignored the influence of impulsive systems, and moreover, researchers called for a more nuanced account of bedtime procrastination from the dual perspective of self-control [9,29]. Thus, based on the above discussion, this study investigates the relationship between self-control and bedtime procrastination from a dual-system perspective.

Meanwhile, previous studies have linked the future time perspective with impulse and control systems. Future time perspective is negatively correlated with impulsivity [30] and positively associated with self-control ability [31]. Notably, impulsivity and self-control have been shown to mediate the effects of future time perspective on mental health [32], internet addiction, and procrastination [18]. The self-regulatory framework of the time perspective [11] has been proposed to explain this mediating effect. The model suggests that individuals with a higher future time perspective have better outcomes because they have better self-control. Specifically, future-oriented individuals are better able to control their behavior and can make more rational rather than impulsive decisions to engage in planned and purposeful behavior. Hence, we propose the second hypothesis:

**Hypothesis** **2** **(H2).** *Impulse and control systems mediate the negative relationship between future time perspective and bedtime procrastination.*

### 1.3. Problematic Smartphone Use as a Mediator

The TST also emphasizes the effect of behavioral prepotency on health behavior. Behavioral prepotency is the frequency of past performance or the presence of cues to action in the environment, which is dictated directly by internal biological drives, salient environmental cues, and past behavior [8]. Past behavior is one of the strongest predictors of future behavior. For example, problematic smartphone use (especially at night) has been widely proven to be a powerful behavioral clue leading to sleep problems [33,34].

Problematic smartphone use, also referred to as smartphone addiction, is a recurrent craving to use a smartphone that is difficult to control and leads to impaired daily functioning [35]. Problematic smartphone use has become widespread and has various unfortunate consequences such as lack of sleep, depression, anxiety, and even suicide [36]. Additionally, previous studies have linked problematic smartphone use with procrastination, suggesting that problematic smartphone use is positively correlated with trait procrastination [37], specific work procrastination [38], and academic procrastination [39]. Similarly, problematic smartphone use was also a predictor of bedtime procrastination [40] and led to poor sleep quality through the mediation of bedtime procrastination [7,41].

Additionally, the orientation of the future as a protective factor against substance use and addictive behavior have been demonstrated in many studies [42]. Similarly, problematic smartphone use is negatively associated with future time perspective [43] and mediates the effect of future time perspective on procrastination [44]. Thus, it is reasonable to expect that problematic smartphone use is also a psychological mechanism of future time perspective affecting bedtime procrastination. Furthermore, the self-regulatory framework of the time perspective may also be used to explain the possible mechanism of this mediating pathway. In addition to self-control ability, the self-regulatory framework of the time perspective also holds that people with a high future time perspective are more likely to experience positive outcomes because they have more excellent goal monitoring and operation [11]. In other words, after establishing behavioral intentions and goals, future-focused people are more likely to monitor their progress toward their goals, adjust possible deviation behavior (problematic smartphone use), and engage in actions and behavior directed toward achieving their goals. Accordingly, we posit the third hypothesis:

**Hypothesis** **3** **(H3).** *Problematic smartphone use mediates the negative relationship between future time perspective and bedtime procrastination.*

### 1.4. Serial Mediating Effect of Dual-Mode Self-Control and Problematic Smartphone Use

Dual-system models have also been used to account for the etiology of the addictive behavior [12]. From this perspective, addictive behavior results from the imbalance between relatively automatic impulsive processes and a somewhat controlled process; this model has also been confirmed in problematic smartphone use, as evidenced by the fact that impulsivity and self-control ability are among the strongest prospective predictors [45,46]. On the one hand, higher impulsivity is a risk factor for problematic smartphone use [47] and the underlying mechanism of the relationship between PTSD and problematic smartphone use [48]. On the other hand, the protective role of self-control on problematic smartphone use has been demonstrated in many studies [49,50]; however, self-regulatory fatigue leads to problematic smartphone use by pursuing pleasure [51]. Accordingly, when people are faced with temptation, if they tend to succumb to the satisfaction of desires, they may be prone to addictive behavior. Contrastingly, if they have enough ability and resources to monitor, inhibit, and regulate themselves, they will move toward rational behavior. In conclusion, by combining the dual-system model of problematic smartphone use and the above evidence, we posit the last hypothesis:

**Hypothesis** **4** **(H4).** *Dual-mode self-control and problematic smartphone use sequentially mediate the negative relationship between future time perspective and bedtime procrastination.*

## 2. Method

### 2.1. Participants

From October to November 2021, a cluster sample of 4162 students from seven schools (one middle school, three high schools, and three universities) in six provinces of China was used. After excluding incomplete questionnaires, the effective sample comprised 3687 participants, ages 11 to 23 (*M* = 16.17, *SD* = 2.42), including 1133 (30.73%) middle school students, 1213 (32.90%) high school students, and 1336 (36.23%) college students. Female participants (*n* = 2110, 57.23%) outnumbered male participants (*n* = 1428, 38.73%), and 149 (4.04%) had missing reports.

### 2.2. Measures

#### 2.2.1. Future Time Perspective

Future time perspective was measured using the future dimensions of the Chinese version [52] of the Zimbardo Time Perspective Inventory (ZTPI) (it consists of five subscales assessing past positive, past negative, present impulsive, present fatalistic, and future) [15]; it contains five items rated on a 5-point Likert scale ranging from 1 (strongly disagree) to 5 (strongly agree) (e.g., I complete projects on time by making steady progress). High scores are indicative of high levels of future time perspective. In the present study, the CFA suggested a good model fit (χ^2^/*df* = 2.44, GFI = 1.00, CFI = 1.00, IFI = 1.00, NFI = 1.00, RMSEA = 0.02, SRMR = 0.01), and Cronbach’s α for the scale was 0.73.

#### 2.2.2. Dual-Mode of Self-Control

The dual-mode self-control was measured using the Chinese version [53] of the dual-mode self-control scale (DMSC-S) [27]; it is a 21-item questionnaire that assesses the control system (e.g., item 9, “I do something to try to solve the problem”, Cronbach’s α = 0.86) and impulse system (e.g., item 12, “I speak without thinking things out”, Cronbach’s α = 0.83) rated on a 5-point Likert scale, ranging from 1 (strongly disagree) to 5 (strongly agree). A high score indicates greater self-control or impulsivity. In the present study, the CFA suggested a good model fit (χ^2^/*df* = 16.69, GFI = 0.92, CFI = 0.91, IFI = 0.91, NFI = 0.90, RMSEA = 0.07, SRMR = 0.05).

#### 2.2.3. Problematic Smartphone Use

Problematic smartphone use was measured using the Chinese version [54] of the Smartphone Addiction Scale-Short Version (SAS-SV) [55]; it contains 10 items rated on a 6-point Likert scale ranging from 1 (strongly disagree) to 6 (strongly agree) (e.g., “using my smartphone longer than I had intended”). High scores indicated high levels of excessive smartphone use. In the present study, the CFA suggested an acceptable model fit (χ^2^/*df* = 36.51, GFI = 0.94, CFI = 0.93, IFI = 0.93, NFI = 0.93, RMSEA = 0.10, SRMR = 0.06), and Cronbach’s α for the scale was 0.87.

#### 2.2.4. Bedtime Procrastination

Bedtime procrastination was measured using the Chinese version [56] of the Bedtime Procrastination Scale (BPS) [2]; it contains nine items rated on a 5-point Likert scale ranging from 1 (never) to 5 (always) (e.g., “I do not go to bed on time”). Higher scores indicated more BP. In the present study, the CFA suggested an acceptable model fit (χ^2^/*df* = 33.12, GFI = 0.94, CFI = 0.92, IFI = 0.92, NFI = 0.91, RMSEA = 0.09, SRMR = 0.05), and Cronbach’s α for the scale was 0.85.

### 2.3. Procedure

The study was permitted by the Ethics Committee of the Faculty of Psychology, Southwest University in China. Informed consent was obtained from the participants and legal guardians before data collection. All participants were informed about the study’s aims and assured that their data would only be used for research purposes. Participants completed questionnaires in their school classrooms, and trained researchers guided the participants when they required assistance. The questionnaires took approximately 20 min to complete. After completing the survey, the questionnaires were collected on the spot.

### 2.4. Data Analysis

In this study, statistical analyses were performed using SPSS 25.0 and the Hayes SPSS macro-PROCESS 3.3 (IBM: Armonk, NY, USA) which is specifically designed to examine sophisticated models involving multiple mediating variables [57]. The analyses were conducted in two steps. Firstly, we calculated descriptive statistics and correlation analysis for study variables. Secondly, we performed hypothesis testing using the PEOCESS macro (Model 80). The bootstrapping methods to test the indirect effects from 5000 random samples, using a 95% confidence interval. If the 95% confidence interval excludes zero, it means that the effects are statistically significant. All variables were standardized to obtain standardized beta coefficients.

## 3. Results

### 3.1. Preliminaryl Analysis

The descriptive statistics and Pearson’s correlations for the study variables are presented in Table 1. All variables were significantly correlated with each other.

Besides, we divided the participants into early adolescence (middle school students), middle adolescence (high school students), and late adolescence (college students). Then, we conducted an independent-sample *t*-test for gender, and conducted a one-way ANOVA for age group (see Table 2). The results revealed that there were no significant gender differences in future time perspective, impulse systems, and control system, whereas gender differences existed in problematic smartphone use and bedtime procrastination. Specifically, females reported significantly higher problematic smartphone use scores (*t* = 6.54, *p* < 0.001) and bedtime procrastination scores (*t* = 5.32, *p* < 0.001) than males. Notably, the age group differences existed in all study variables. Specifically, the level of future time perspective was significantly higher in late adolescence than middle adolescence (*p* < 0.01) and early adolescence (*p* < 0.001), and the early adolescence was significantly higher than middle adolescence (*p* < 0.001). The level of impulse system was significantly lower in late adolescence than early adolescence (*p* < 0.01), but there were no significant differences between middle adolescence with early adolescence (*p* > 0.05) and late adolescence (*p* > 0.05). The level of control system and problematic smartphone use was significantly higher in late adolescence than early adolescence (*p* < 0.001) and middle adolescence (*p* < 0.001), but there was no significant difference between middle adolescence and early adolescence (*p* > 0.05). Finally, the level of bedtime procrastination was significantly lower in early adolescence than middle adolescence (*p* < 0.001) and late adolescence (*p* < 0.001), but there was no significant difference between middle adolescence and late adolescence (*p* > 0.05). Therefore, we included gender and grade as covariates in subsequent analyses to obtain pure effects.

### 3.2. Testing the Multiple Mediation Model

**Figure 1:**
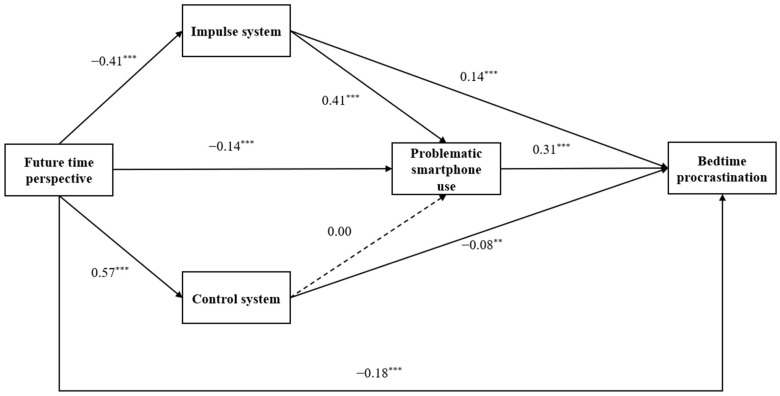
The serial mediation effect of dual-mode self-control and problematic smartphone use in the relationship between future time perspective and bedtime procrastination (standardized coefficients.). Note. Gender and grade were included as covariates, although the paths and coefficients are not displayed. ** *p* < 0.01, *** *p* < 0.001.

We utilized Model 80 of the PROCESS macro to examine the mediating effect of dual-model self-control and problematic smartphone use on the relationship between future time perspective and bedtime procrastination. Table 3 and Figure 1 present the results of the mediation model. After accounting for the effects of covariates, the negatively predictive effect of future time perspective on bedtime procrastination was significant (*β* = −0.38, *p* < 0.001); moreover, when dual-model self-control and problematic smartphone use were considered mediators, future time perspective negatively predicted impulse system (*β* = −0.41, *p* < 0.001) and problematic smartphone use (*β* = −0.14, *p* < 0.001), and positively predicted control system (*β* = 0.57, *p* < 0.001). In turn, the impulse system had positively predictive effects on problematic smartphone use (*β* = 0.41, *p* < 0.001) and bedtime procrastination (*β* = 0.14, *p* < 0.001), the control system had negatively predictive effect on bedtime procrastination (*β* = −0.08, *p* < 0.01) but did not significant predict problematic smartphone use (*β* = 0.00, *p* > 0.05). Meanwhile, problematic smartphone use had positively predictive effect on bedtime procrastination (*β* = −0.31, *p* < 0.001). Furthermore, when the mediators were added, the direct effect of future time perspective on bedtime procrastination was still significant (*β* = −0.18, *p* < 0.001). Finally, the bootstrap results indicated the significant indirect effect of dual-mode self-control and problematic smartphone use on the relationship between future time perspective and bedtime procrastination, the total indirect effect was −0.20 (*p* < 0.001, 95% *CI* = [−0.23, −0.17]), and the ratio of the indirect effect to the total effect was 52.63%. Specifically, the mediating effect consisted of four paths: the sole mediating effect of impulse system (*β* = −0.06, 95% *CI* = [−0.07, −0.04]), control system (*β* = −0.04, 95% *CI* = [−0.07, −0.02]), and problematic smartphone use (*β* = −0.05, 95% *CI* = [−0.06, −0.03]), and the sequential mediating effect of impulse system and problematic smartphone use (*β* = −0.05, 95% *CI* = [−0.06, −0.03]); however, the sequential mediating effect of the control system and problematic smartphone use was not significant (*β* = 0.00, 95% *CI* = [−0.01, 0.01]). The ratio of the four indirect effects to the total effect were 15.79%, 10.53%, 13.16%, and 13.16%.

## 4. Discussion

Regarding health behavior, the present study takes TST as the framework, integrates the self-regulatory model of time perspective and dual-process models of addictive behavior, and examines the effects of future time perspective (temporal valuations), dual-mode self-control (self-regulatory capacity), and problematic smartphone use (behavior prepotency) on bedtime procrastination. The results demonstrated that bedtime procrastination was related to future time perspective, dual-mode self-control, and problematic smartphone use and that dual-mode self-control and problematic smartphone use partly account for the link between future time perspective and bedtime procrastination. Specifically, future-oriented individuals have higher self-control and fewer impulsive decisions, which prevents excessive smartphone use, reducing the tendency to delay bedtime.

### 4.1. Future Time Perspective as a Predictor of Bedtime Procrastination

This study documented future time perspective as an essential predictor of bedtime procrastination, thus supporting H1. Our findings are consistent with previous studies, which showed that future time perspective plays a protective role in procrastination [19] and health behavior [20,21]. Based on the TST [8], we can explain this in terms of valence and temporal proximity. Future-focused people have a higher expected value for long-term beneficial goals [13] and are more aware of the value of regular sleep for their health and function. Furthermore, they fully consider the potential link between current behavior and distant outcomes [14] and perceive themselves as moving forward from the present moment into the future, which makes the connection between the present and the future closer. Thus, the higher value-rated and perceived temporal proximity of sleep prompted individuals with higher future time perspective to have stronger sleep intentions and go to bed on time. The meta-analysis results that found that future time perspective facilitates behavioral purposes also support this inference [16].

### 4.2. Mediating Roles of Dual-Mode Self-Control and Problematic Smartphone Use

Moreover, our study showed that impulse and control systems mediated the relationship between future time perspective and bedtime procrastination, thus supporting H2; these findings are consistent with the self-regulatory framework of the time perspective [11]. Future time perspective may affect self-control ability (including impulsivity and control), affecting behavioral outcomes. Future-oriented individuals tend to place greater importance on long-term, distal motives, which lead to higher self-control [31] and lower impulsivity [30]. Subsequently, even if self-regulatory resources are depleted in the evening, they will also exert excellent self-regulation before going to bed because of their superior control ability and rational decision-making, preventing bedtime procrastination [2,5,7]. One previous study confirmed that far-reaching goal orientation and behavioral components independently predicted bedtime procrastination through self-control’s mediating effect and provided some evidence for these findings [58].

Similarly, we found that problematic smartphone use mediated the relationship between future time perspective and bedtime procrastination, thus supporting H3. The self-regulatory framework of the time perspective was also used to explain the possible mechanism of this mediating pathway; this model maintains that the future time perspective is associated with better outcomes via the self-regulatory process of goal monitoring and operation [11]. Specifically, after establishing sleep intentions, future-focused people are more likely to regularly monitor their behavior, engage in more sleep-promoting behaviors, and reduce excessive smartphone use, thus avoiding bedtime procrastination caused by smartphone use [6,40].

Finally, our study also demonstrated that the association between future time perspective and bedtime procrastination could be sequentially mediated by the impulse system and problematic smartphone use rather than the control system and problematic smartphone use, partially supporting H4; these findings are consistent with previous studies on impulsivity predicting problematic smartphone use [47] but contradict the results that self-control prevents individuals from relying on smartphones [50]. Once established, one possible explanation is that addiction is perpetuated by robust impulsive processes but receives little control from reflective processes [12]. Specifically, individuals with high future time perspective have fewer impulsive tendencies, so they can protect themselves from the temptation to overuse their smartphones without excessive volitional control, thereby ensuring the successful execution of sleep goals. Consequently, bedtime procrastination is reduced.

### 4.3. Implications and Limitations

This study has important theoretical and practical implications for bedtime procrastination research. Theoretically, in contrast to the procrastination perspective, this study, based on the health behavior perspective, proposed that BP was caused by the failure of “intention-behavior” transformation and because of the weak sleep intention caused by people’s low perceived temporal valuations of sleep. Conversely, future-focused people have strong sleep intentions because of their higher attachment to the benefits of distant sleep; thus, they go to bed on time rather than procrastination. Second, in contrast to studies that focused only on the control dimension, the present study examined the relationship between self-control and bedtime procrastination from a dual system of control and impulsivity to provide a deeper understanding of the relationship. Finally, under the self-regulatory model of time perspective, this study hypothesized and confirmed that self-control and problematic smartphone use played a mediating rather than a moderating role between intentions and health behaviors, which was different from the TST perspective; this result enlightens the researchers to look into the relationship between behavioral intention, self-control, behavioral prepotency, and health behaviors from a different perspective. Practically, these results contribute to preventing and intervening in adolescent bedtime procrastination. First, because those who have lower future time perspective are more likely to delay sleep, educators should pay attention to the students’ developmental levels of future time perspective and help to develop their future thinking and planning skills which could decrease the likelihood of becoming a bedtime procrastinator. Second, our study clarified the mediating roles of dual-mode self-control in the relationship between future time perspective and bedtime procrastination; this enlightens educators to not only focus on cultivating students’ self-control ability, but also on reducing impulsivity when developing prevention and intervention programs. Finally, this study also found the important role for problematic smartphone use. Therefore, parents and teachers should strengthen their control over students’ smartphone use, especially to reduce their use time at night, so as to ensure health sleep.

Although this study has some highlights, several limitations must be considered. First, all study variables were subject to self-reporting bias. Second, although theoretical and empirical studies support the causal chain of our model, our cross-sectional design allows us to draw tentative conclusions about causal relationships. In particular, TST points out that behavioral outcomes may affect temporal valuations, self-control capacity, and behavioral prepotency via the feedback loop [8]. Thus, the potential reciprocity between study variables should be tested in future studies. Finally, future time perspective is usually defined as a multidimensional concept [14], and future research needs to examine the effects of different dimensions in detail.

## 5. Conclusions

In sum, our results support future time perspective as a protective factor for adolescent bedtime procrastination. Most importantly, future time perspective can indirectly make an impact on bedtime procrastination through the single mediating effects of impulse system, control system, and problematic smartphone use, and the chain mediating effect, by decreasing the level of impulsivity, thereby reducing excessive smartphone use; this health behavior perspective of bedtime procrastination provides new possibilities for understanding and addressing its harmful health effects. While current study has certainly answered some research questions, it has also thrown up many more that need further investigation. For example, knowledge about the contributing factors and potential solutions to bedtime procrastination needs to be further deepened.

## Figures and Tables

**Table 1:** Descriptive statistics and correlations among variables.

Variable	*M*	*SD*	FTP	IS	CS	PSU	BP
FTP	3.51	0.66	-				
IS	2.42	0.68	−0.41 ***	-			
CS	3.44	0.60	0.58 ***	−0.42 ***	-		
PSU	3.35	0.98	−0.30 ***	0.47 ***	−0.24 ***	-	
BP	3.20	0.74	−0.37 ***	0.39 ***	−0.31 ***	0.46 ***	-

Note. FTP, future time perspective; IS, impulse system; CS, control system; PSU, problematic smartphone use; BP, bedtime procrastination. *** *p* < 0.001.

**Table 2:** Gender and age group difference among variables.

Variable	Female (2110)	Male (1428)	*t*	EA (1133)	MA (1213)	LA (1341)	*F*
FTP	3.51 ± 0.64	3.52 ± 0.69	−0.36	3.54 ± 0.69	3.36 ± 0.65	3.62 ± 0.61	52.11 ***
IS	2.45 ± 0.66	2.40 ± 0.70	1.92	2.48 ± 0.73	2.44 ± 0.65	2.38 ± 0.64	6.17 **
CS	3.42 ± 0.57	3.45 ± 0.64	−1.63	3.39 ± 0.65	3.38 ± 0.57	3.51 ± 0.56	19.94 ***
PSU	3.43 ± 0.96	3.21 ± 1.01	6.54 ***	3.21 ± 1.05	3.30 ± 0.98	3.52 ± 0.91	33.69 ***
BP	3.25 ± 0.74	3.11 ± 0.73	5.32 ***	3.12 ± 0.76	3.23 ± 0.73	3.24 ± 0.72	9.92 ***

Note. FTP, future time perspective; IS, impulse system; CS, control system; PSU, problematic smartphone use; BP, bedtime procrastination; EA, early adolescence; MA, middle adolescence; LA, late adolescence. ** *p* < 0.01; *** *p* < 0.001.

**Table 3:** Hierarchical multiple regression results of the effects of future time perspective on bedtime procrastination through dual-mode self-control and problematic smartphone use.

	BP		IS		CS		PSU		BP	
	*β*	*t*	*β*	*t*	*β*	*t*	*β*	*t*	*β*	*t*
Gender	−0.07	−4.72 ***	−0.04	−2.26 *	0.04	−2.89 **	−0.07	−4.55 ***	−0.04	−2.84 **
Grade	0.06	3.87 ***	−0.03	−1.67	0.07	5.10 ***	0.14	9.38 ***	0.03	2.06 *
FTP	−0.38	−24.40 ***	−0.41	−26.70 ***	0.57	41.84 ***	−0.14	−7.96 ***	−0.18	−9.98 ***
IS							0.41	25.17 ***	0.14	8.33 ***
CS							0.00	0.14	−0.08	−4.22 ***
PSU									0.31	19.00 ***
R^2^	0.15		0.17		0.34		0.26		0.30	
F	211.71 ***		242.31 ***		602.80 ***		249.73 ***		248.42 ***	

Note. FTP, future time perspective; IS, impulse system; CS, control system; PSU, problematic smartphone use; BP, bedtime procrastination. * *p* < 0.05, ** *p* < 0.01, *** *p* < 0.001.

## Data Availability

Data is available from the corresponding author upon reasonable request.
